# The Role of miRNAs as Predictors of Acute Lymphoblastic Leukemia Chemotherapy Toxicity in Children: A Systematic Review

**DOI:** 10.3390/jcm14165869

**Published:** 2025-08-20

**Authors:** Małgorzata M. Mitura-Lesiuk, Maciej Dubaj, Karol Bigosiński, Mateusz Raniewicz

**Affiliations:** 1 Department of Pediatric Hematology, Oncology and Transplantology, Medical University of Lublin, 6 Gębali Str., 20-093 Lublin, Poland; 2 Doctoral School, Medical University of Lublin, 20-093 Lublin, Poland; maciej.dubaj99@gmail.com (M.D.); karolbig145a@wp.pl (K.B.); 3 Student Scientific Association, Department of Pediatric Hematology, Oncology and Transplantology, Medical University of Lublin, 20-093 Lublin, Poland; mateusz.raniewicz.mr@gmail.com

**Keywords:** acute lymphoblastic leukemia, children, miRNAs, chemotherapy complications, toxicity

## Abstract

**Background/Objectives**: Acute lymphoblastic leukemia (ALL) is the most common childhood cancer, accounting for 80% of leukemias in this group and about 25% of all cancers. The 5-year survival rate is now over 90%. Achieving such a good outcome is made possible by the introduction of intensive, high-dose chemotherapy. However, it is associated with numerous complications, affecting up to 80% of patients. Among the most common of these are infections and intestinal, hepatic, hematological or neurological complications. For their effective treatment and prevention, it is necessary to develop predictors. High hopes in this aspect are placed on miRNAs. The aim of the following paper is to present the role of miRNAs as predictors of chemotherapy complications in children with ALL. **Methods**: A systematic review of the available literature in the PubMed, Scopus, Embase and Google Scholar scientific databases was conducted. Fourteen publications were included in the analysis. **Results**: Changes in miRNA expression and single-nucleotide polymorphisms in miRNAs are associated with complications of ALL therapy. Among the most notable are miR-1206 (in mucositis and myelotoxicity), miR-2053 (in neurotoxicity and mucositis), miR-938 and miR-3117 (in gastrointestinal toxicity and neurotoxicity), miR-1307 (in gastrointestinal toxicity and mucositis) and miR-323b (in gastrointestinal toxicity and myelotoxicity). In addition, miR-155, miR-3117 and miR-4268 may be potential therapeutic targets in complications of ALL therapy. **Conclusions**: miRNAs are good potential predictors of ALL chemotherapy toxicity and may be therapeutic targets in these complications.

## 1. Introduction

Acute lymphoblastic leukemia (ALL) is the most common childhood malignancy, accounting for 75–80% of leukemia cases in this group [1,2,3]. Its estimated annual global prevalence is 285,095, an increase of approximately 60% from 1990 [1,2,3]. The opposite trend is observed for mortality, which has declined by nearly 70% over this period [1,2,3]. In developed countries, the five-year survival rate is 90–96% [3,4,5]. Such good rates are gained by introducing new therapeutic approaches, based on multidrug chemotherapy and hematopoietic stem cell transplantation (HSCT), as well as new methods like targeted therapies [3,4].

Treatment of ALL is usually long-lasting, with a duration of up to two years [5]. Despite differences in the therapeutic protocols used around the world, they are based on high-dose treatment with steroids, methotrexate, anthracyclines, vincristine, L-asparaginase, cyclophosphamide, etoposide, 6-mercaptopurine and cytarabine [5]. However, their use is associated with a relatively high rate of therapy-related complications (TRCs) of even more than 80% [6,7]. TRCs can be divided into early ones, developing up to two weeks after the start of therapy, and late ones, developing after the end of treatment [7]. Acute complications mainly include infections (68–88%), gastrointestinal (diarrhea, vomiting, constipation, toxic liver damage and stomatitis) (27–36%), neurological (4–27%) and endocrine (4–15%) complications, and thrombotic incidents (8.5–10%) [6,7]. Distant complications include arrhythmias and heart failure, hypertension, chronic obstructive pulmonary disease, cognitive impairment, osteoporosis and increased risk of bone fractures, as well as secondary cancer [8]. In order to effectively treat and prevent these complications, it is crucial to detect them early and even predict them, which is possible with predictors. Those with proven efficacy include patient age, patient race, obesity, baseline leukocyte count, blood type, history of kidney disease, higher levels of drug metabolites and gene polymorphisms (for example, *TPMT*, *NUDT15*, *MTHFR*, *ABCB1/2* and *TYMS*) [9,10,11,12,13,14,15,16,17]. High hopes in this field have been attached to the use of miRNAs.

MiRNAs are non-coding RNAs that play a role in gene expression [18]. They are involved in the pathogenesis and progression of cancer and may also be potential prognostic and predictive factors [18,19]. Some studies show that they can also be used as predictors of chemotherapy toxicity in cancer, including childhood acute lymphoblastic leukemia [19,20]. There are systematic reviews about the use of miRNAs as markers of side effects of specific drugs, such as methotrexate, or complications, such as neurological ones [20,21,22,23,24]. However, there is a lack of reviews that capture the described issue holistically.

The main aim of this paper is to present the role of miRNAs as predictors of chemotherapy complications in children treated for acute lymphoblastic leukemia.

## 2. Materials and Methods

A systematic review of the literature available in the scientific databases PubMed, Google Scholar, Scopus and Embase from 2014 to 2024 was conducted. The review was conducted from 5 May to 1 July 2025. We were looking for scientific articles that described the role of miRNAs as potential predictors of acute and distant complications of chemotherapy in children treated for acute lymphoblastic leukemia using standard treatment protocols. The present paper was not registered and was not submitted to the Bioethics Committee due to the nature of the systematic review, the lack of experimental character of the study and the fact that such studies are generally accepted by local bioethics committees. The search was conducted by two reviewers simultaneously, then the information they found was synthesized and the resulting discrepancies resolved. Neither reviewer had a conflict of interest, both being independent researchers, which eliminated potential risks. The reviewers had adequate knowledge of pediatric ALL and miRNAs and experience in conducting a systematic review. By employing two reviewers, the potential risk of bias in the selection of publications and assessment of conclusions was minimized. The following keywords were used: ‘miRNAs’, ‘chemotherapy toxicity’, ‘chemotherapy complications’, ‘therapy complications’, ‘acute lymphoblastic leukemia’, ‘child’ and ‘children’, using the logical operator ‘AND’. The inclusion criteria for the review were as follows: studies written in the English language only, the nature of the paper (original research paper, systematic review or meta-analysis), the period of publication of the article (2014–2024), papers on acute lymphoblastic leukemia only and papers on patients under 18 years of age only. The exclusion criteria were as follows: papers written in a language other than English; papers in the form of letters to the editor, abstracts or editorials; papers on other characteristics of miRNAs than as predictors of chemotherapy complications (such as prognostic and predictive factors); papers on diseases other than acute lymphoblastic leukemia; and papers on adult patients. Non-full-text papers were excluded from the review due to the frequent lack of demographic and clinical descriptions of the study groups and detailed descriptions of the methodology and research apparatus used. They also often lack a description of the target genes for miRNA. Both reviewers collected their data, which were then jointly analyzed. Differences in their results consisted of the inclusion of additional papers, such as those that included both pediatric and adult populations, which were eliminated after joint discussion. The collected results were presented using the odds ratio (OR) and 95% confidence interval (CI) or the area under the curve (AUC) from the receiver operating characteristic (ROC) curve.

**Figure 1:**
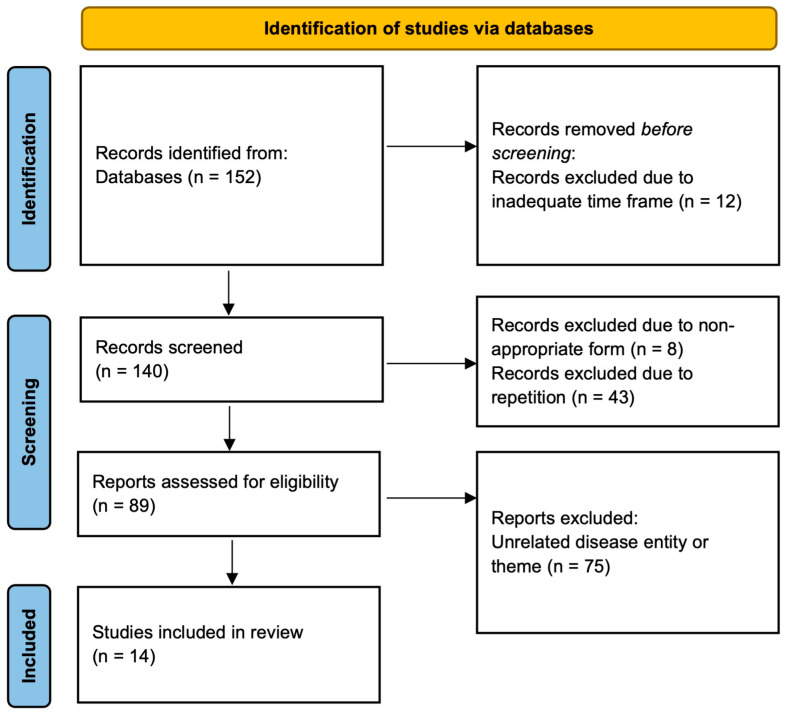
The PRISMA flowchart showing the methodology of systematic review.

Initially, the titles and abstracts of the articles were analyzed, resulting in 140 articles. After rejecting duplicate articles, 97 were left. Next, articles incompatible in nature with the inclusion criteria were eliminated from the analysis (89 left), followed by a substantive analysis of the publications, rejecting 75 of them. Finally, 14 publications were included in the review. The PRISMA flowchart of the paper search is shown in Figure 1 [25].

## 3. Results

### 3.1. Mucositis

Mucositis, especially oral mucositis, is one of the most common complications occurring in pediatric patients undergoing ALL treatment. It affects 18–40% of children treated with chemotherapy and up to 80% of children undergoing HSCT [24,26,27,28]. It occurs mainly in boys aged 5–7 years, more often with malnutrition [24,26,27]. In addition to reduced quality of life and worsening of complaints, it may be associated with reduced treatment efficacy [24,27].

López-López et al. in a group of 152 B-ALL patients in the consolidation phase of therapy (LAL-SHOP 94/99/2005 protocols) observed that single-nucleotide polymorphisms (SNPs) in miR-1206 (rs2114358), miR-2053 (rs10505168), miR-1307 (rs7911488) and miR-146a (rs2910164) were associated with a higher risk of developing mucositis in children with ALL. This was mainly relevant for miR-1206 (odds ratio [OR]: 4.90; 95% confidence interval [CI]: 1.49–20.21), which through its target genes *ABCC2* and *ABCG2* affects methotrexate (MTX) metabolism [29,30,31]. The second most important was miR-1307 (OR: 2.80; 95%CI: 1.04–7.54), which affects cell sensitivity to cisplatin through its effect on *MDM4* and *MCF7* expression [29,30,31].

Gutierrez-Camino in a group of 117 patients with ALL noted that the SNP rs2114358 in miR-1206 was significantly associated with a higher risk of oral mucositis in patients treated with MTX (OR: 3.6; 95%CI: 1.1–11.5). This complication occurred in 18.8% of patients [32]. It supports previous observations, especially considering the involvement of miR-1206 in MTX metabolism and toxicity [30]. According to a systematic review by Umerez et al., SNPs in this miRNA are the most promising markers of mucositis in children [21]. In another study, Gutierrez-Camino et al. (*n* = 170) made an interesting observation. They noted that SNPs of miR-3683, miR-4520a and miR-1908 were significantly associated with a higher incidence of mucositis, and SNP rs4674470 in miR-4268 was significantly associated with a lower incidence, which may be some kind of protective factor [33]. The target genes of the described miRNAs are involved in the metabolic pathways of drugs used in ALL chemotherapy, including *NFKBIE* and *CBR1* in daunorubicin metabolism and MTHFR, *MTR* and *SLC46A1* in MTX metabolism [33]. Meanwhile, the PLD signaling pathway, which is also targeted by miR-4268, has an important role in mucositis in inflammatory bowel diseases, and *PLD* silencing reduces the secretion of inflammatory cytokines and inhibits mucosal inflammation [34]. Therefore, the above miRNA may be a good therapeutic target for mucositis in children with ALL [33]. The role of miRNAs as predictors of mucositis as a complication of ALL therapy in children is shown in Table 1.

### 3.2. Gastrointestinal Toxicity

Gastrointestinal complications are also a major problem during ALL treatment. They occur in up to 67.1% of patients [35]. Gutierrez-Camino et al. pointed out that diarrhea occurs in almost 13% of patients in the induction phase and 6% in the consolidation phase, while vomiting occurs in 26.5% and 24%, respectively [33]. The same authors noted that SNP rs8667 in miR-4751 is associated with a higher risk of diarrhea (OR: 12.83; 95%CI: 1.67–98.8) and rs12402181 in miR-3117 with a lower incidence of vomiting (OR: 0.24; 95%CI: 0.08–0.72) [33]. Mir-4751 is involved in the TLR signaling pathway, which acts pro-inflammatory by stimulating the expression of IL-1, Il-6 or TNF [30]. Researchers have also confirmed the role of this pathway in the induction of inflammatory bowel diseases, as well as diarrhea, by triggering intestinal mucosal inflammation and affecting the gut microbiota composition [36,37]. Involvement of miR-3117 in the pro-inflammatory MAPK pathway also confirms the observed results. Inhibition of the pathway’s activity reduces the pain sensation, as well as diarrhea and vomiting, which may be, and in some cases (aprepitant) already is, a therapeutic target [38]. López-López et al. in turn observed that SNPs in miR-453 (OR: 2.9; 95%CI: 1.23–6.82) and miR-323b [the synonym of miR-492] (OR: 3.75; 95%CI: 1.16–12.08) were significantly associated with a higher incidence of vomiting during the induction phase of therapy, miR-1307 (OR: 4.55; 95%CI: 1.31–15.72) with a higher incidence of diarrhea, and miR-423 (OR: 0.28; 95%CI: 0.1–0.83) with a lower risk of developing this intestinal complication [29]. The described SNP in miR-453 is particularly relevant, as it has been shown to be associated with MTX clearance (OR: 2.27; 95%CI: 1.08–4.77) [29]. Its target genes are ABCC1, ABCB1, ABCC2 and ABCC4, which encode trans-membrane transporters involved in MTX metabolism and are also involved in its excretion through the intestine and urethra [39,40]. Polymorphisms of these genes are responsible for changes in the clearance of MTX, its accumulation in the blood and increase the risk of side effects [40].

In a study by da Silva Menezes et al. in 77 patients with B-ALL, it was observed that rs2505901 in miR-938 and rs56103835 in miR-323b were significantly associated with a lower risk of intestinal toxicity of therapy (OR: 0.2; 95%CI: 0.04–0.96; OR: 0.23; 95%CI: 0.05–0.96, respectively) [35]. The above observation is consistent with the observation in the same population, where de Souza et al. indicated a protective role of miR-938 against the development of ALL in children [41]. An association of miR-938 with the development of gastric cancer, colorectal cancer or Hirschsprung’s disease has also been observed [42,43]. In turn, miR-323b is associated with genes encoding the above-mentioned transporters in MTX metabolism, affecting its blood concentration [29]. The role of miRNAs as predictors of gastrointestinal toxicity as a complication of ALL therapy in children is shown in Table 2.

### 3.3. Hepatic Toxicity

Another major complication, particularly relevant to drug metabolism, is hepatic injury [44,45]. According to some researchers, it may be the most common treatment-related toxic complication, with rates ranging from 23% to as high as 66.5% [44,45]. According to Gutierrez-Camino et al., it occurs in 32.3% of patients in the induction phase and 29.2% in the consolidation phase [46]. During the induction phase, it can be associated with the administration of asparaginase, and in the later stages of treatment with MTX [47]. In the aforementioned study (*n* = 179) among patients with B-ALL, one observed that the rs264881 variant in miR-1208 was significantly associated with a lower risk of hepatotoxicity (OR: 0.11; 95%CI: 0.02–0.46), including severe hepatotoxicity, from grade 3 and above [46]. The authors also noted an association between hepatotoxicity and the presence of variants in miR-4707, miR-3698d2, miR-300, miR-5197 and miR-3936 in the induction phase and miR-3615, miR-3144, miR-4745, miR-4467, miR-5189, miR-1908, miR-5197 and miR-4472-1 in the consolidation phase, but these results were not statistically significant after the false discovery rate implementation [46]. The target genes for miR-1208 were those involved in MTX metabolism, including *DHFR*, *MTHFR* and *SLCO1A2* [46]. Interestingly, higher expression of *DHFR*, *MTHFR* and *MTR* genes was associated with inhibition of hepatocyte apoptosis, showing a protective effect [46,48]. Moreover, miR-1208 also targets genes related to 6-mercaptopurine metabolism (*TPMT*, *ABCC5* and *NT5C2*) [46]. Their higher expression may lead to increased inactivation of the drug, reduced thiopurine concentrations in the hepatocyte and thus a protective effect on liver cells [49].

López-López et al. observed that the rs12894467 variant in miR-300 was significantly associated with the occurrence of hepatotoxicity (OR: 5.12; 95%CI: 1.63–16.16) and hyperbilirubinemia (OR: 4.4; 95%CI: 1.37–14.13). In addition, the rs34115976 variant in miR-577 was associated with lower prevalence of hyperbilirubinemia in patients with ALL (OR: 0.27, 95%CI: 0.08–0.98) [29]. Among miR-300 target genes, those responsible for neutralizing vincristine in hepatocytes (*ABCC1*, *ABCB1*) and cyclophosphamide (*ALDH5A1*) are prominent. Their reduced expression caused by SNPs in miRNAs can result in increased drug toxicity and impact on the liver [29,44].

Esmaili et al. also made an interesting observation in their study of 74 patients undergoing ALL treatment in the consolidation phase and 41 healthy controls. The expression level of miR-24 was not significantly different between patients with and without hepatotoxicity. However, it was significantly lower in patients than in healthy controls (*p* < 0.004). In addition, the expression of this miRNA was significantly decreased in patients with a higher severity of hepatic toxicity (grade II–IV) after MTX treatment who were hospitalized for complications or had their MTX dose discontinued or reduced (*p* = 0.025) [50]. Reduced miR-24 expression in other studies was found in patients with ALL, and overexpression of this miRNA was associated with MTX resistance and increased toxicity [51,52,53,54]. The role of miRNAs as predictors of hepatotoxicity as a complication of ALL therapy in children is shown in Table 3.

### 3.4. Cardiotoxicity

A major complication of ALL treatment, especially the use of anthracyclines and mediastinal radiotherapy, is cardiotoxicity [55]. It is estimated that it can affect up to 60% of pediatric oncology patients [56]. Among the recognized and applicable predictors and markers of cardiotoxicity are troponin T (cTnT), C-reactive protein (CRP), IL-6, IL-37 and galectin-3 levels [57]. Moreover, numerous gene polymorphisms are also prominent predictors, including rs2229774 in *RARG*, rs3743527 in *ABCC1* or rs17863783 in *UGT1A6* [57]. The role of such predictors is also explored among miRNAs.

Leger et al. in a group of 37 subjects (25 treated with anthracyclines) observed altered expression of three miRNAs in treated patients compared to controls. Measurements were made at 6, 12 and 24 h after administration of the drug. After 6, 12 and 24 h, miR-1 and miR-499 expression was significantly higher in patients compared to controls, while miR-29b expression was higher only after 6 h. Moreover, miR-29b and miR-499 levels were significantly correlated with cTnT levels after 6 and 24 h, respectively. MiR-29b at 6 h and miR-499 at 6 and 24 h were characterized by area under the curve (AUC) = 0.74, AUC = 0.82 and AUC = 0.9, respectively, in distinguishing patients with cardiotoxicity from those who had no toxicity. An even better result (AUC = 0.9) was achieved when both markers were combined 6 h after drug administration [58]. The abovementioned miRNAs are specific for muscle, especially cardiomyocytes, and show high expression after myocardial infarction, being markers of acute myocardial injury [59,60]. In addition, the miR-29 family is able to aggravate infarct-related damage by inhibiting the PI3K/mTOR/HIF1α/VEGF pathway, so it may also represent a potential therapeutic target in this disease [60].

Cheung et al. in a group of patients with ALL (*n* = 32) and acute myeloid leukemia (AML) (*n* = 7) observed that circulating serum miR-1 and cTnT are good markers of myocardial injury after anthracyclines with AUC = 0.62 (95%CI: 0.38–0.97) and AUC = 0.62 (95%CI: 0.4–0.84), respectively [61]. The above observations have been confirmed in studies among adult patients [62,63]. Mir-1 is directly involved in cardiogenesis and the recovery of myocardial cells after injury (via *IRX5*, *KCNE1*, *SLC8A1*, *B56A*, *ACTA2* and *MYH11*) [64].

**Figure 2:**
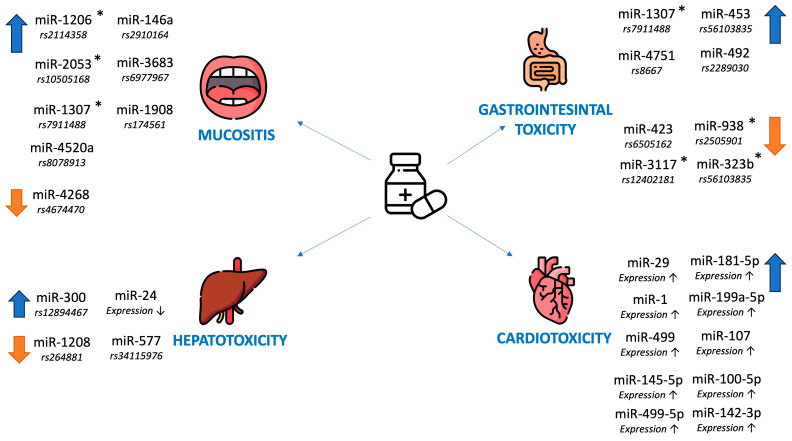
The role of miRNAs as predictors of mucositis, gastrointestinal, hepatic and cardiotoxicity as a complication of ALL therapy in children; ↑ = higher risk, ↓ = lower risk; * = the molecule is mentioned in two independent studies.

Oatmen et al. in a group of 20 patients with pediatric cancer (they did not specify the exact diagnosis) observed a significant correlation between altered expression of miR-181-5p, miR-199a-5p, miR-107, miR-499-5p, miR-145-5p, miR-100-5p, miR-103a-3p and miR-142-3p and a decrease in LVEF >10% during anthracycline treatment [65]. The clinical utility of miR-29 and miR-499 was also confirmed by Antoniadi et al. in their review [66]. The role of miRNAs as predictors of cardiotoxicity as a complication of ALL therapy in children is shown in Table 4. The role of miRNAs as predictors of mucositis and gastrointestinal, hepatic and cardiac toxicity is summarized in Figure 2.

### 3.5. Renal Toxicity

Renal complications of ALL therapy occur in approximately 4% of children in the induction phase and 10% in the consolidation phase [29]. These include acute kidney injury (AKI), most commonly caused by MTX administration and occurring in up to 20% of patients receiving CAR-T therapy [29,67,68]. López-López observed that rs10061133 in miR-449b (OR: 11.1; 95%CI: 1.71–71.9) and rs2368393 in miR-604 (OR: 4.15; 95%CI: 1.07–16.15) were significantly associated with the occurrence of renal complications [29]. Qin et al. in a rat model study observed that cisplatin administration increases miR-449 expression in proximal tubule cells, which enhances their damage and death by regulating the Sirt1/p53/Bax pathway [69]. Moreover, both miRNAs are associated with increased inflammatory response and renal damage in the mechanism of AKI [70].

### 3.6. Myelotoxicity

Hematologic toxicity occurs in up to 89.1–91.6% of ALL patients during the maintenance phase of treatment [71,72]. Anemia and neutropenia affect about half of patients, while thrombocytopenia is less common (about 6%) [73]. Most often, myelotoxicity is mild—of grade 1 or 2—but grade 3 and 4 toxicity affects up to 52.8–54.9% [73,74]. This is mainly due to the use of MTX and 6-mercaptopurine [73,74]. It is a major complication, through which a reduction in the chemotherapeutic dose is necessary [69,70]. Grade 4 myelotoxicity in the induction phase of therapy occurs in 60% (neutropenia), 34% (anemia) and 51% (thrombocytopenia) of patients, respectively [75]. This contributes to a high incidence of infections in this group, affecting 60–86% of patients [35,76]. Interestingly, infections remain the leading (72%) cause of death in children during the induction phase [77]. Predictors of myelotoxicity in children with ALL include polymorphisms in the *TMPT*, *ITPA* and *NUDT15* genes [78], as well as some of the miRNAs.

Da Silva Menezes noted that myelotoxicity occurred in 62.8% of subjects, and infections, the most common complication, occurred in up to 85.7% [35]. They observed that SNPs rs12904 in miR200c (OR: 0.26; 95%CI: 0.73–0.92), rs3746444 in miR-499a (OR: 0.23; 95%CI: 0.06–0.83) and rs10739971 in let7a1 (OR: 0.18; 95%CI: 0.04–0.93) were associated with a lower risk of myelotoxicity in patients treated for ALL. Moreover, rs2043556 in miR-605 was associated with a significantly lower incidence of infections as a complication (OR: 0.08; 95%CI: 0.01–0.54) [35]. Decreased expression of miR-200c is one of the potential diagnostic markers in childhood ALL, whose target genes are *ABCA2* and *ABCA3*, whose expression is in turn affected by MTX [79]. In turn, the aforementioned SNP in miR-499 is also a diagnostic marker in ALL and AML [80]. Its role in the pathogenesis of B-ALL is based on the regulation of gene expression: *FOXO1A* (bone marrow cell differentiation, higher risk of relapse in ALL), *MS4A1* (encoding B-lymphocyte marker—CD20) and *PBX1* (involved in ALL pathogenesis, unfavorable prognosis, association with hyperleukocytosis) [80,81,82,83]. The let-7 family also has a role in the pathogenesis of ALL in children. Their decreased expression leads to increased expression of *NRAS*, *HMG2A* and *MYC* proto-oncogenes [84]. MiRNAs from the let-7a-1 cluster are furthermore specific for human hematopoietic cells and are involved in hematopoiesis [85].

Zhan et al. made observations in a group of 181 patients with ALL who received a total of 654 cycles of MTX chemotherapy in the consolidation phase [30]. Leukopenia occurred in 98% of cycles in this group, and anemia occurred in 96.48% of cycles. They noted that SNP rs2114358 in miR-1206 was significantly associated with grade 3/4 leukopenia and rs56103835 in miR-323b with grade 3/4 anemia in MTX-treated patients [30]. The target genes for miR-1206 were *SLCO1A2*, *ABCC2*, *ABCG2*, *TYMS* and *FPGS*, while those for miR-323 were *ABCC1*, *ABCC2*, *ABCC4* and *SHTM1* [30]. These are genes encoding transporters or enzymes involved in the pharmacodynamics of MTX, which can affect the level of the drug in the blood and therefore explain its toxicity [30,86]. In addition, polymorphisms of the aforementioned genes are also predictors of chemotherapy toxicity, such as rs1045642 in the *ABCB1* gene associated with neutropenia or rs717620 in *ABCC2* increasing the risk of pancytopenia in children treated with MTX [87]. The above miRNAs thus represent promising biomarkers of severe MTX myelotoxicity. The role of miRNAs as predictors of myelotoxicity as a complication of ALL therapy in children is shown in Table 5.

### 3.7. Neurotoxicity

Neurotoxicity of drugs used to treat ALL is a significant problem, complicating patients’ functioning and limiting their prognosis. Some authors even suggest that neurotoxicity is the most common complication of ALL treatment [88]. Among the main neurological complications are vascular incidents, subacute leukoencephalopathy, acute encephalopathy, syndrome of inappropriate antidiuretic hormone secretion and aseptic meningitis [89]. The syndromes depend on the applied drug. Hence, aseptic meningitis, stroke-like syndrome and leukoencephalopathy are characteristic of MTX, affecting 3-11% of children with ALL [88,89,90]. For cytarabine, cerebellar damage is characteristic; for asparaginase, thromboembolic incidents; and for vincristine, peripheral polyneuropathy [88,89,90]. Among the known predictors of chemotherapy-induced neurotoxicity in ALL are high serum creatinine levels, Hispanic ethnicity, liver dysfunction, older age (>10 years), high doses of MTX, female gender and T-ALL leukemia type [16,88,90,91].

Chen et al. conducted a study on young rats receiving treatment for childhood acute leukemia and analyzed the effects of MTX on the nervous system. They observed increased expression of miR-155 in the rats’ plasma at 24 h after MTX administration. Moreover, anti-miR-155 played an important role in maintaining the integrity of the blood–brain barrier by increasing the expression of claudin-1 as a barrier component. Through this, it has a protective function against the influence of MTX and reducing cognitive impairment [92]. Decreased expression of this miRNA has previously been indicated as a potential neuroprotective factor in inflammatory and ischemic brain conditions [93]. This observation represents a milestone, outlining possibilities for future research using miRNAs as therapeutic targets.

Gutierrez-Camino et al. in a population of 179 children with ALL observed the occurrence of neurotoxicity in 175 of them, with 85.5% of events during the induction phase [94]. They observed that rs12402181 in miR-3117 was associated with a reduced risk of neurotoxicity, while rs7896283 in miR-4481 was associated with more than a twofold increased risk of vincristine-induced toxicity [94]. Among the target genes for miR-3117, the authors identified *ABCC1* and *RALBP1*, which upregulate the elimination of vincristine from cells, which would explain the reduced risk of neurotoxicity in this group [22,94]. On the other hand, miR-4881 is involved in the axon guidance pathway, which determines the proper formation of neuronal connections and cognitive functions [95].

Martin-Guerrero et al., in a group of 152 Spanish children treated with vincristine, found that rs12355840 in miR-202 was associated with a significantly higher incidence of grade 1–2 neurotoxicity (OR: 2.88; 95%CI: 1.07–7.72) [96]. The target for this miRNA was the *TUBB2B* gene, involved in neuronal structure [96,97]. Low-grade neurotoxicity accompanied 19.1% of patients in the induction phase and 3.3% in later phases [96]. The above results, although statistically significant, were considered by the author to be weak in clinical significance. However, they provide a clue for the development of further studies.

Da Silva Menezes et al. in a Brazilian population of children with ALL reported the incidence of neurotoxicity to be 22.8%. They observed that rs2292832 in miR-149 (OR: 7.26; 95%CI: 1.44–36.54), rs10505168 in miR-2053 (OR: 4.61; 95%CI: 1.21–17.65) and rs2043556 in miR-605 (OR: 10.23; 95%CI: 1.12–93.34) were associated with an increased risk of neurotoxicity in children. Moreover, rs2505901 in miR-938 significantly reduced the risk of this complication (OR: 0.09; 95%CI: 0.01–0.79) [35]. miR-149 is involved in modulating the inflammatory response, carcinogenesis and sensitivity to treatment in other diseases, including neuroblastoma [98,99].

**Figure 3:**
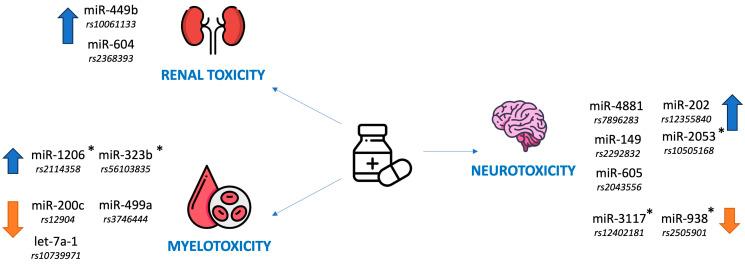
The role of miRNAs as predictors of renal, hematological and neurological toxicity as a complication of ALL therapy in children; ↑ = higher risk, ↓ = lower risk; * = the molecule is mentioned in two independent studies.

**Figure 4:**
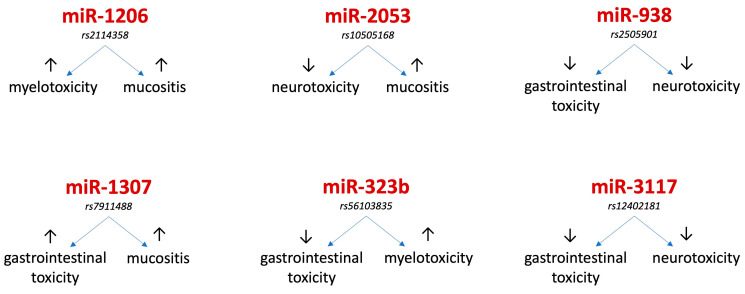
miRNAs that play a predictor role in two different complications of ALL therapy; ↑ = higher risk, ↓ = lower risk.

It is also worth remembering that some miRNAs (miR-181a and miR-181c-5p) are implicated in the development of nervous system involvement in ALL and are good diagnostic markers of it [100,101]. The role of miRNAs as predictors of neurotoxicity as a complication of ALL therapy in children is shown in Table 6. A summary of the role of miRNAs as predictors of renal, hematologic and neurologic toxicity is shown in Figure 3. Figure 4 shows miRNAs that play a role independently in two different complications of ALL therapy.

In the described studies, the principles for determining SNPs or the expression levels of relevant miRNAs were the same—quantitative reverse transcription polymerase chain reaction (RT-qPCR), mainly TaqMan assay. Northern blot, in situ hybridization and microarray methods were used. Of all these, PCR is considered the gold standard [102]. However, the apparatus used differed. The aim of introducing miRNA into diagnostic protocols would be primarily to standardize measurement methods and calibrate measurements appropriately. Research shows that miRNAs have enormous potential for future use as standard diagnostic methods. They possess characteristics that are considered ideal for biomarkers: increasing availability, high sensitivity and specificity [102]. It is essential that the results obtained are reproducible in subsequent trials [102,103]. In the context of our review, miR-1206 and miR-323b, described in various studies by different teams, stand out in this respect. Unfortunately, the reproducibility of these results is still too low to conclude that they are absolutely effective. Certain variables are also unknown, such as differences in levels, interfering factors (other diseases, including inflammation) or modulating parameters such as gender and age, which are particularly important in pediatric patients [104]. It is also important to remember that miRNA expression is altered in the disease itself, through changes in the activity of enzymes, including Drosha, as well as transcription errors [105]. Although the results of studies on miRNAs, including those presented in this review, are extremely promising for the future, it is necessary to clarify their functions. In order to be able to use miRNAs widely, it is necessary to conduct studies on sufficiently large groups, preferably prospective studies with a longer observation period. The next step will be to validate the miRNA detection and measurements methods so that they are standardized. It should also be remembered that the subject of miRNA use is relatively new (since 2008), and the research results are promising and definitely worth further exploration [106].

**Limitations of the study:** This paper draws on only a small number of publications due to the few studies conducted to date. The studies were conducted in small groups of children, mostly in single-center models, and involved the use of various therapeutic regimens. Due to the very small number of publications, an assessment of the strength of the evidence was not conducted, all of which are presented. The study groups differed essentially only in the regimens used and were of similar size and age. All of the groups included a population of children under 18 years of age.

## 4. Conclusions

Both miRNA expression alterations and SNPs in miRNAs are potential good predictors of chemotherapy toxicity in children treated for ALL. Of particular interest may be miR-1206, miR-2053, miR-1307, miR-3117, miR-938 and miR-323b, which are associated with more than one complication of ALL therapy. Their use may influence the delineation of risk groups and determine appropriate management. In addition, some of them, such as miR-155, miR-3117 and miR-4268, may be potential therapeutic targets for particular complications of chemotherapy. However, further randomized studies on large, heterogeneous groups of patients are needed to confirm these observations.

## Figures and Tables

**Table 1:** The role of miRNAs as predictors of mucositis as a complication of ALL therapy in children [29,32,33].

Research	Country	Study Group	Treatment	Phase	miRNA	Changes	Risk	Targets
López-López et al. (2014) [29]	Spain	N = 152 B-ALL	LAL-SHOP 94/99/2005	Cons	miR-1206	SNP rs2114358	↑	*SLCO1A2* *ABCC2* *ABCG2*
					miR-2053	SNP rs10505168	↑	*FPK* *PI3K/AKT*
					miR-1307	SNP rs7911488	↑	*BMPR2* *MDM4* *MCF7* Bcl2
					miR-146a	SNP rs2910164	↑	*TRAF6* *IRAK1*
Gutierrez-Camino et al. (2017) [32]	Spain	N = 117 T-ALL B-ALL	DCOG ALL10	Cons	miR-1206	SNP rs2114358	↑	*SLCO1A2* *ABCC2* *ABCG2*
Gutierrez-Camino et al. (2018) [33]	Spain	N = 170 B-ALL	LAL-SHOP 94/99/2005	Ind	miR-3683	SNP rs6977967	↑	*SHMT1* *ALDH5A1*
					miR-1908	SNP rs174561	↑	-
					miR-4250a	SNP rs8078913	↑	-
					miR-4268	SNP rs4674470	↓	*NFKBIE* *CBR1* *MTHFR* *MTR* *SLC46A* *PLD*

Abbreviations: N—number; SNP—single-nucleotide polymorphism; Cons—consolidation; Ind—induction; ALL—acute lymphoblastic leukemia; ↑ = higher risk, ↓ = lower risk.

**Table 2:** The role of miRNAs as predictors of gastrointestinal toxicity as a complication of ALL therapy in children [29,33,35].

Research	Country	Study Group	Treatment	Phase	miRNA	Changes	Risk	Targets
**DIARRHEA**								
López-López et al. (2014) [29]	Spain	N = 152 B-ALL	LAL-SHOP 94/99/2005	Ind	miR-1307	SNP rs7911488	↑	*FOXO3A* *SMYD4* *TUSC5* *ING5*
					miR-423	SNP rs6505162	↓	*RFVT3* *SRF*
Gutierrez-Camino et al. (2018) [33]	Spain	N = 170 B-ALL	LAL-SHOP 94/99/2005	Ind	miR-4751	SNP rs8667	↑	*NDUSF2* *SLC19A1* *ERCC4* TLR
**VOMITING**								
López-López et al. (2014) [29]	Spain	N = 152 B-ALL	LAL-SHOP 94/99/2005	Cons	miR-453	SNP rs56103835	↑	*ABCC1* *ABCB1* *ABCC2* *ABCC4*
				Ind	miR-323b [synonym: miR-492]	SNP rs2289030	↑	*CD44* *PTEN* *TIMP2*
Gutierrez-Camino et al. (2018) [33]	Spain	N = 170 B-ALL	LAL-SHOP 94/99/2005	Ind	miR-3117	SNP rs12402181	↓	*ABCC1* *PPAT* *SLC46A1* *SLCO1A2* *ABCC1* *ALDH5A1* *MAPK*
**GASTROINTESTINAL TOXICITY**								
da Silva Menezes et al. (2022) [35]	Brazil	N = 77 B-ALL	ALL IC-BFM 2002	-	miR-938	SNP rs2505901	↓	*CXCL12* *SMAD3* *IL17A* *RBM5*
					miR-323b	SNP rs56103835	↓	*ABCC1* *ABCB1 ABCC2 ABCC4*

Abbreviations: N—number; SNP—single-nucleotide polymorphism; Cons—consolidation; Ind—induction; ALL—acute lymphoblastic leukemia; ↑ = higher risk, ↓ = lower risk.

**Table 3:** The role of miRNAs as predictors of hepatotoxicity as a complication of ALL therapy in children [29,46,50].

Research	Country	Study Group	Treatment	Phase	miRNA	Changes	Risk	Targets
López-López et al. (2014) [29]	Spain	N = 152 B-ALL	LAL-SHOP 94/99/2005	Ind	miR-300	SNP rs12894467	↑	*ABCC1* *ABCB1* *ALDH5A1*
					miR-577	SNP rs34115976	↓	*HOXA1* *WNT2B*
Gutierrez-Camino et al. (2018) [46]	Spain	N = 179 B-ALL	LAL-SHOP 94/99/2005	Cons	miR-1208	SNP rs264881	↓	*DHFR* *MTHFR* *MTR* *SLCO1A2* *SLC46A1*
Esmaili et al. (2020) [50]	Iran	N = 74 B-ALL T-ALL	BFM-2009	Cons	miR-24	Expression ↓	↑ (II–IV)	*FAF1* *APAF1* *MYC* *E2F2* *CCNB1* *CDC2*

Abbreviations: N—number; SNP—single-nucleotide polymorphism; Cons—consolidation; Ind—induction; ALL—acute lymphoblastic leukemia; ↑ = higher risk, ↓ = lower risk.

**Table 4:** The role of miRNAs as predictors of cardiotoxicity as a complication of ALL therapy in children [58,61,65].

Research	Country	Study Group	Treatment	Phase	miRNA	Changes	Risk	Targets
Leger et al. (2017) [58]	USA	N = 37 ALL	anthracyclines	Cons	miR-29b miR-499 miR-1	Expression ↑	↑	PI3K mTOR HIF1α *VEGF*
Cheung et al. (2014) [61]	China	N = 40 ALL AML	anthracyclines	-	miR-1	Expression ↑	↑	*IRX5* *KCNE1* *SLC8A1* *B56A* *ACTA2* *MYH11*
Oatmen et al. (2020) [65]	USA	N = 20	anthracyclines	-	miR-181-5p miR-199a-5p miR-107 miR-499-5p miR-145-5p miR-100-5p miR-103a-3p miR-142-3p	Expression ↓	↑	-

Abbreviations: N—number; USA—United States of America; ALL—acute lymphoblastic leukemia; AML—acute myeloid leukemia; Cons—consolidation; ↑ = higher risk, ↓ = lower risk.

**Table 5:** The role of miRNAs as predictors of myelotoxicity as a complication of ALL therapy in children [30,35].

Research	Country	Study Group	Treatment	Phase	miRNA	Changes	Risk	Targets
da Silva Menezes et al. (2022) [35]	Brazil	N = 77 B-ALL	ALL IC-BFM 2002	-	miR-200c	SNP rs12904	↑	*ABCA2* *ABCA3*
					miR-499a	SNP rs3746444		*FOXO1A* *PBX1*
					let-7a-1	SNP rs10739971		*NRAS* *MYC*
Zhan et al. (2023) [30]	China	N = 181 ALL	GD-2008-ALL SCCLG-2016-ALL	Cons	miR-1206	SNP rs2114358	↑	*SLCO1A2 ABCC2* *ABCG2* *TYMS* *FPGS*
					miR-323b	SNP rs56103835		*ABCC1/2/4* *SHTM1*

Abbreviations: N—number; SNP—single-nucleotide polymorphism; Cons—consolidation; ALL—acute lymphoblastic leukemia; ↑ = higher risk.

**Table 6:** The role of miRNAs as predictors of neurotoxicity as a complication of ALL therapy in children [35,94,96].

Research	Country	Study Group	Treatment	Phase	miRNA	Changes	Risk	Targets
da Silva Menezes et al. (2022) [35]	Brazil	N = 77 B-ALL	ALL IC-BFM 2002	-	miR-149	SNP rs2292832	↑	*CDC42* *BCL2* *SIRT1*
					miR-2053	SNP rs10505168	↑	*KIF3C* PI3K/Akt
					miR-605	SNP rs2043556	↑	*MDM2*
					miR-938	SNP rs2505901	↓	*RBM5* *SMAD3*
Gutierrez-Camino et al. (2018) [94]	Spain	N = 179 B-ALL	LAL-SHOP 94/99/2005	Ind	miR-3117	SNP rs12402181	↓	*ABCC1* *RALBP1*
					miR-4881	SNP rs7896283	↑	Axon guidance pathway
Martin-Guerrero et al. (2019) [96]	Spain	N = 152 B-ALL	LAL-SHOP 94/99/2005	Ind	miR-202	SNP rs12355840	↑ (I–II)	*TUBB2B*

Abbreviations: N—number; ALL—acute lymphoblastic leukemia; SNP—single-nucleotide polymorphism; Ind—induction; ↑ = higher risk, ↓ = lower risk.

## Data Availability

All data are available in the manuscript.

## Abbreviations

The following abbreviations are used in this manuscript:

ALL	Acute lymphoblastic leukemia
OR	Odds ratio
CI	Confidence interval
SNP	Single-nucleotide polymorphism
HSCT	Hematopoietic stem cell transplantation
TRC	Treatment-related complications
MTX	Methotrexate
AKI	Acute kidney injury
AML	Acute myeloid leukemia
AUC	Area under the curve
